# Supplementary figures and images for: Individualized network analysis reveals a link between the gut microbiome, diet intervention and Gestational Diabetes Mellitus
Source: PLoS Comput Biol. 2023 Jun 29;19(6):e1011193. doi: 10.1371/journal.pcbi.1011193 (PMC10337958; doi:10.1371/journal.pcbi.1011193)

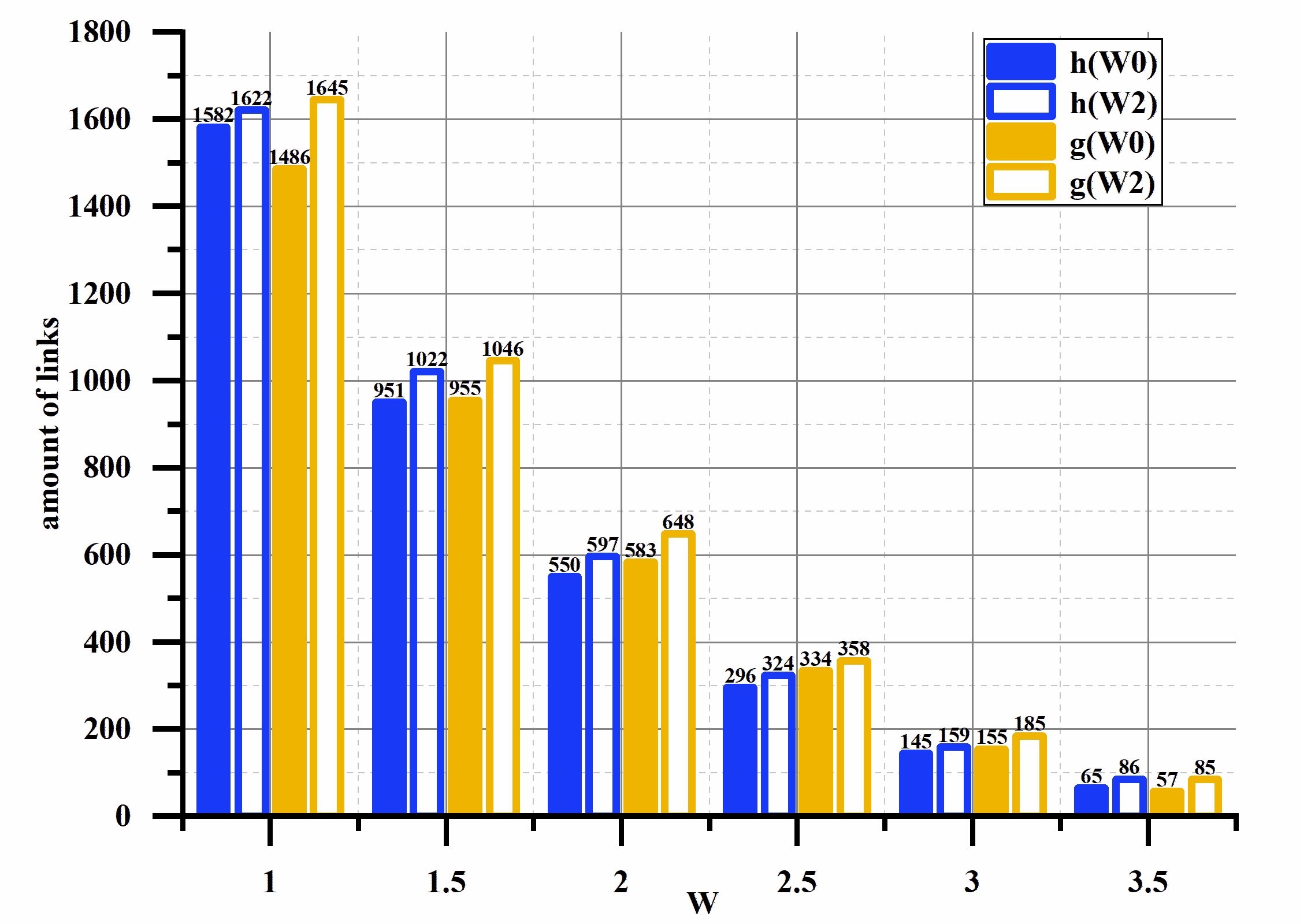

Supplement: S1 Fig — The corresponding network size of different groups is different though the threshold is fixed. (TIF) [file pcbi.1011193.s002.tif]

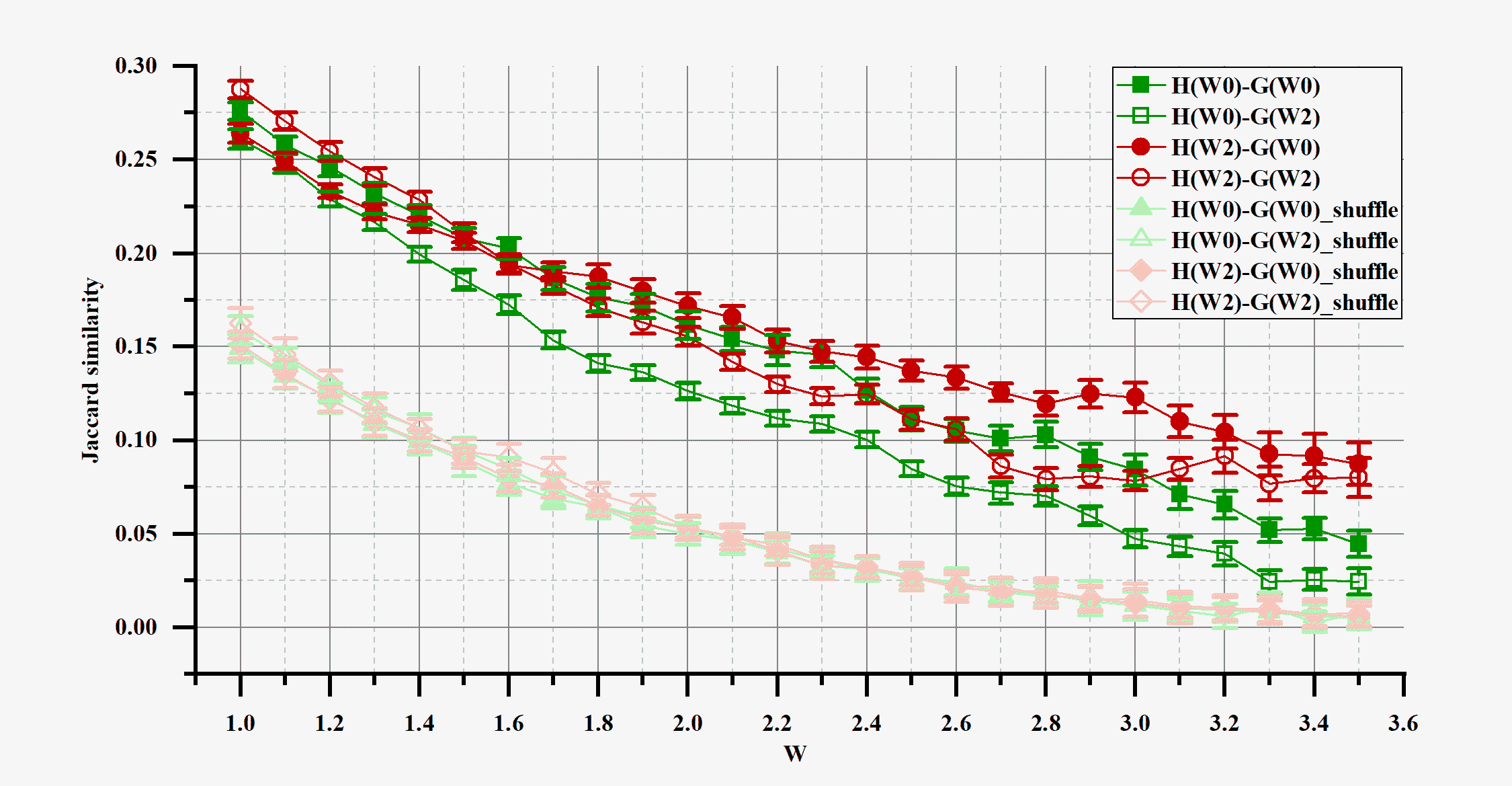

Supplement: S2 Fig — The green curve shows the GDM group compared with the healthy group two weeks earlier, and the red curve shows the GDM group compared with the healthy group two weeks later. The solid dots indicate the comparison between the GDM group and the healthy group before the dietary intervention, and the hollow dots indicate the comparison between the GDM group and the healthy group after the dietary intervention. The dark curve is the real data result, and the light curve is the shuffled network result. (TIF) [file pcbi.1011193.s003.tif]

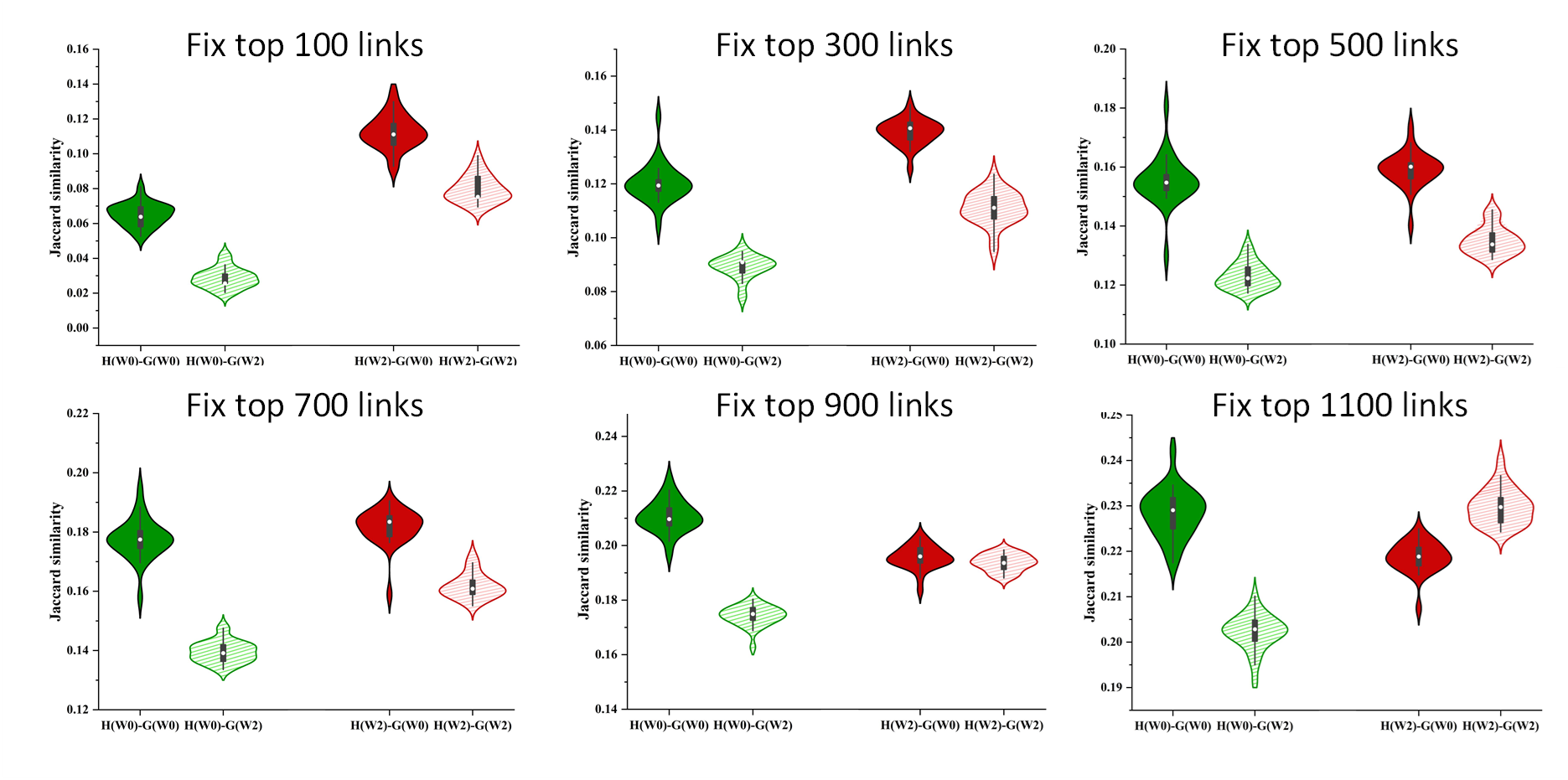

Supplement: S3 Fig — When different number of links are fixed, the pattern is still stable in most cases. (TIF) [file pcbi.1011193.s004.tif]
